# Molecular characterization of *Wolbachia* infection in bed bugs (*Cimex lectularius*) collected from several localities in France

**DOI:** 10.1051/parasite/2016031

**Published:** 2016-08-05

**Authors:** Mohammad Akhoundi, Arnaud Cannet, Céline Loubatier, Jean-Michel Berenger, Arezki Izri, Pierre Marty, Pascal Delaunay

**Affiliations:** 1 Service de Parasitologie-Mycologie, Hôpital de l’Archet, Centre Hospitalier Universitaire de Nice 06000 Nice France; 2 Inserm U1065, Centre Méditerranéen de Médecine Moléculaire, Faculté de Médecine, Université Nice-Sophia Antipolis 06000 Nice France; 3 Faculté de Médecine, Université Nice-Sophia Antipolis 06000 Nice France; 4 URMITE, UM63, CNRS 7278, IRD 198, Inserm 1095, Faculté de Médecine, Université Aix-Marseille 13000 Marseille France; 5 Service de Parasitologie-Mycologie, Hôpital Avicenne, Assistance Publique-Hôpitaux de Paris 93000 Bobigny France

**Keywords:** Bed bugs, *Cimex lectularius*, Symbiont, *Wolbachia*

## Abstract

*Wolbachia* symbionts are maternally inherited intracellular bacteria that have been detected in numerous insects including bed bugs. The objective of this study, the first epidemiological study in Europe, was to screen *Wolbachia* infection among *Cimex lectularius* collected in the field, using PCR targeting the surface protein gene (*wsp*), and to compare obtained *Wolbachia* strains with those reported from laboratory colonies of *C. lectularius* as well as other *Wolbachia* groups. For this purpose, 284 bed bug specimens were caught and studied from eight different regions of France including the suburbs of Paris, Bouches-du-Rhône, Lot-et-Garonne, and five localities in Alpes-Maritimes. Among the samples, 166 were adults and the remaining 118 were considered nymphs. In all, 47 out of 118 nymphs (40%) and 61 out of 166 adults (37%) were found positive on *wsp* screening. Among the positive cases, 10 samples were selected randomly for sequencing. The sequences had 100% homology with *wsp* sequences belonging to the F-supergroup strains of *Wolbachia*. Therefore, we confirm the similarity of *Wolbachia* strains detected in this epidemiological study to *Wolbachia* spp. reported from laboratory colonies of *C. lectularius*.

## Abstract (fr)

Les symbiotes *Wolbachia* sont des bactéries intracellulaires maternellement héritées qui ont été détectés chez de nombreux insectes, y compris les punaises de lit. Les objectifs de cette étude, la première étude épidémiologique en Europe, était de cribler l’infection par *Wolbachia* chez les *Cimex lectularius* recueillis sur le terrain, en utilisant la PCR ciblant le gène de la protéine de surface (*wsp*) et de comparer la souche de *Wolbachia* obtenue avec celles rapportées des colonies de laboratoire de *C. lectularius* et d’autres groupes de *Wolbachia*. Dans ce but, 284 spécimens de punaises de lit ont été capturés et étudiés, provenant de huit régions différentes de la France, y compris les banlieues de Paris, les Bouches-du-Rhône, le Lot-et-Garonne et cinq localités dans les Alpes-Maritimes. Parmi les échantillons, 166 étaient des adultes et le reste (118) ont été considérés comme des nymphes. Quarante-sept des 118 nymphes (40 %) et 61 des 166 adultes (37 %) ont été trouvés positifs pour le dépistage de *wsp*. Parmi les cas positifs, 10 échantillons ont été choisis au hasard pour le séquençage. Les séquences avaient 100 % d’homologie avec des séquences de *wsp* appartenant aux souches du supergroupe F de *Wolbachia*. Par conséquent, nous confirmons la similitude des souches de *Wolbachia* détectées dans cette étude épidémiologique à celles des *Wolbachia* spp. signalées dans les colonies de laboratoire de *C. lectularius*.

## Introduction

Bed bugs are blood-sucking insects and human ectoparasites, and have re-emerged over the last two decades worldwide. In economically advanced countries, they are a serious public health issue that still threaten many people. They may cause problems in housing facilities, public facilities, and residential complexes. They are therefore an important risk factor that cannot be neglected by the tourism industry, the housing industry, and public institutions. Bed bug infestations have been reported to have physical and psychological effects in humans. Despite isolation of several pathogens, found in the bed bug body, they have not been confirmed as a vector of pathogens to humans [18]. Recently, Salazar et al. [32] and Leulmi et al. [15] reported the vectorial competence of *C. lectularius* for *Trypanosoma cruzi* and *Bartonella quintana* transmissions in laboratory experiments. In addition, bed bug infections by *Burkholderia multivorans* have been reported by Saenz et al. [28], who screened different bed bug populations in the United States.

A wide range of symbiotic microorganisms are associated with insect hosts that mainly reside in their guts, body cavities, or cells [4, 13, 19]. Some obligate symbionts with a mutualistic nature – like *Buchnera* in aphids and *Wigglesworthia* in tsetse – have been reported to contribute to host fitness [9, 22], whereas some other symbionts such as *Wolbachia* are often parasitic causing negative effects in their hosts [24]. The latter seem to be obligate commensal symbionts and facultative parasites that are of importance due to their roles in forces driving the evolutionary transition into mutualism [8, 10].

*Wolbachia* are maternally inherited rickettsia-like bacteria (Gram-negative alpha-proteobacteria), harbored by a wide range of arthropods and are mainly found in the host gonads [5, 23, 26, 35]. These intracellular bacteria were first reported in 1924 in the reproductive tissues of *Culex pipientis* mosquitos [11]. Since this discovery, several papers have reported the presence of these bacteria in the host tissues of many different arthropod species including bed bugs. This presence and association of *Wolbachia* in hosts is mainly considered as a commensal or somewhat parasitic relationship. The *Wolbachia* infection often leads to host reproductive phenotypes, such as cytoplasmic incompatibility, feminization, male-killing, anti-virus properties, parthenogenesis, increased or decreased fitness, and sperm-egg incompatibility, which consequently results in unfertilized eggs [6, 8, 16, 20, 34, 37]. In addition to the roles mentioned, *Wolbachia* have been shown to occupy bacteriocytes (specialized cells that constitute a pair of organs known as bacteriomes) and to be essential for bedbug growth and reproduction via provisioning of B vitamins [14, 21]. Moreover, *Wolbachia* increase male bed bug fitness by increasing the egg laying rate of the female after mating. The bacterium also leads to an increase in female host fitness by increasing egg hatch and development success, increasing offspring body size, and reducing fitness costs (wounding and infection) associated with traumatic insemination [13].

Several *Wolbachia* strains have been reported from different arthropod species. They have been classified in eight major supergroups from A to H. *Wolbachia* species belonging to A, B, and E groups infect diverse arthropods; C and D infect nematodes; G infects spiders and H infects termites, whereas F infects arthropods and nematodes [3, 21, 25, 27, 29–31, 34]. Infections with *Wolbachia* species of F supergroup seem to be prevalent in the Cimicinae subfamily (*Cimex* and *Oeciacus* genera). The species appears to be monophyletic, suggesting that *Wolbachia* symbionts were first introduced to a primitive insect population and then diverged dependently, along with the insect hosts in this subfamily.

Despite this knowledge on the spread of *Wolbachia* infection among different arthropod hosts including bed bugs, the contribution rate of *Wolbachia* in field collected bed bug populations has not been studied sufficiently in Europe.

Therefore, the aim of the present study was to screen the prevalence of *Wolbachia* sp. that colonize naturally field collected populations of *Cimex lectularius* using a PCR-based survey, and to compare obtained *Wolbachia* strains with those that have been reported from laboratory colonized *C. lectularius* as well as other *Wolbachia* groups.

## Materials and methods

**Figure 1.**
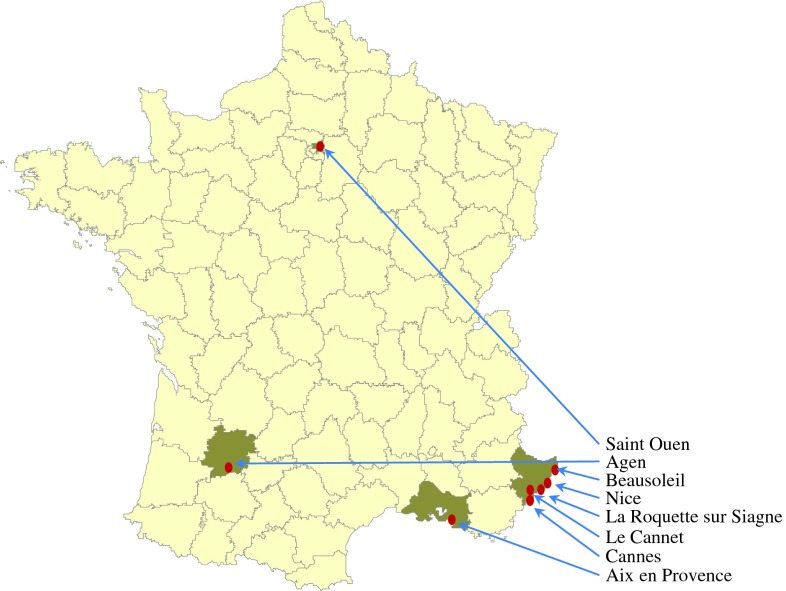
Collect sites of bed bug (*C. lectularius*) populations in France.

*Cimex lectularius* specimens were collected from four different parts of France, including hotel and individual apartments from 2010 to 2012. They were caught from the suburbs of Paris (Saint-Ouen), Bouches-du-Rhône (Aix-en-Provence), Lot-et-Garonne (Agen), and Alpes-Maritimes (Nice, Cannes, Le Cannet, Beausoleil, and La Roquette-sur-Siagne) (Fig. 1). All samples within infested structures were collected from a single room. All bed bugs (except the egg stage) were hand-removed by entomological tweezers during inspections, transferred immediately into the labeled microtubes containing ethanol 96%, and stored at −20 °C for further molecular analysis.

DNA was extracted using Qiagen’s DNeasy extraction kit (Valencia, CA, USA) according to the manufacturer’s protocol. Moreover, the purity and concentration of extracted DNA was measured using a NanoDrop spectrophotometer (BioTek Synergy 2, USA). The samples were then screened for the presence of *Wolbachia* species*.* For this purpose, specific primers for *wsp*, 81F (F: 5′-TGG TCC AAT AAG TGA TGA AGA AAC-3′) and *wsp* 691R (R: 5′-AAA AAT TAA ACG CTA CTC CA-3′), were used for 600 bp fragment amplification of *Wolbachia* surface protein (*wsp*) by polymerase chain reaction [36]. A pair of positive (*Wolbachia* positive samples previously sequenced for confirmation) and negative (DNA free distilled water) controls were used in each PCR run. Following amplification, the samples were fractionated via horizontal submerged gel electrophoresis using 1.5% agarose gels with DNA size markers (Promega PCR markers), and obtained DNA fragments were visualized by ethidium bromide staining before sequencing. PCR products were sequenced in both directions directly, using the *wsp* 81F and *wsp* 691R primers used for DNA amplification. The sequences were then edited using Staden package software and compared to homologous sequences in GenBank thanks to the nucleotide Basic Local Alignment Search Tool (BLAST: www.ncbi.nlm.nih.gov/BLAST) in order to identify the samples at the species level. Strains were considered as identified at the species level when their sequence showed ≥99% homology with a sequence deposited in GenBank. Sequences were also aligned with BioEdit v7.0.0 software [12] for comparison.

## Results

**Figure 2.**
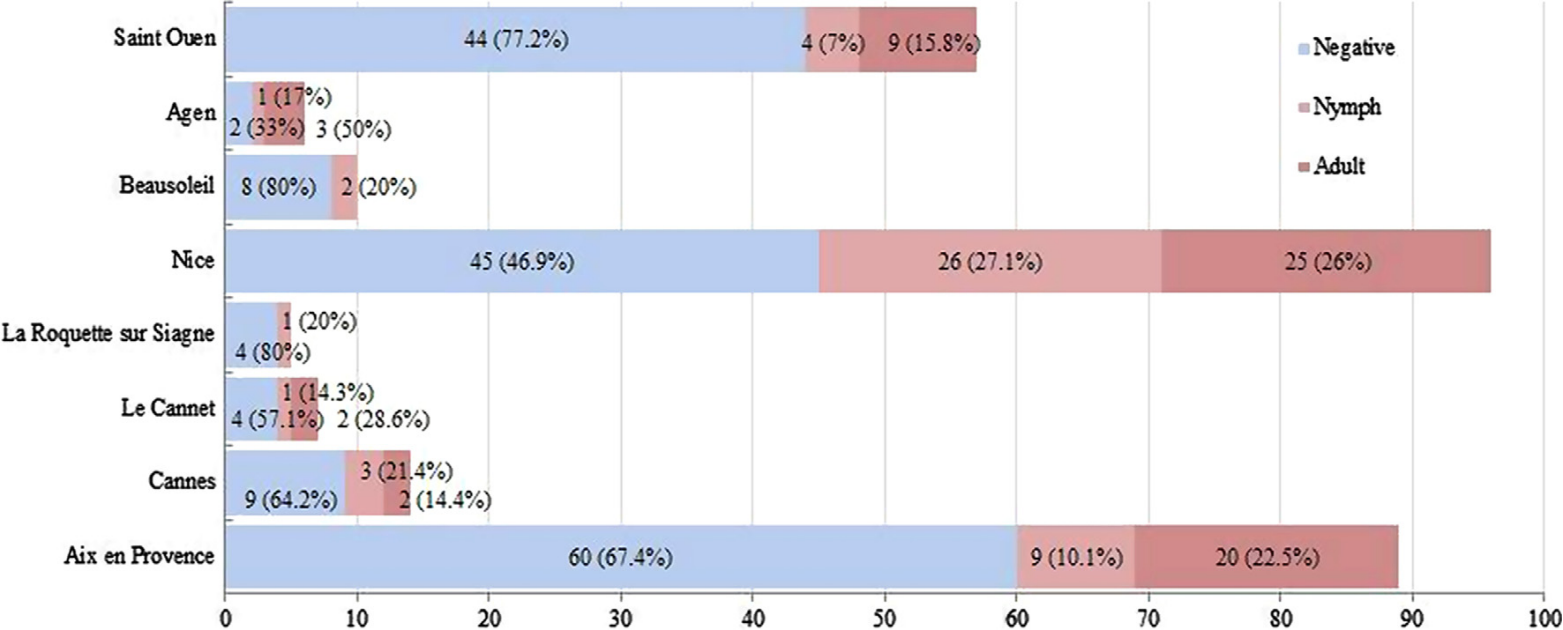
Number (percent) positivity for *Wolbachia* in adult and nymph bed bugs in different regions.

In the present study, the prospected samples were demonstrated to have suitable DNA quality using Nanodrop spectrometry and electrophoresis gel running. In total, 284 bed bug specimens were captured from eight sites: Aix-en-Provence, Saint-Ouen, Nice, Beausoleil, Le Cannet, Cannes, La Roquette-sur-Siagne, and Agen. They were screened to detect the *Wolbachia wsp* gene. The largest number of samples was from Nice (96 samples) followed by Aix-en-Provence (89 samples), and the lowest number of samples analyzed in this study was from La Roquette-sur-Siagne (4 samples). Among all samples, 166 were adults and the remaining samples (117) were considered nymphs (Fig. 2, Table 1).

**Table 1.** *Cimex lectularius* populations prospected for *Wolbachia* infection in the present study.

District	Bouches-du-Rhône		Suburbs of Paris		Alpes-Maritimes										Lot-et-Garonne		Total
City	Aix-en-Provence		Saint-Ouen		Nice		Beausoleil		Le Cannet		Cannes		La Roquette-sur-Siagne		Agen		
*C. lecturius*	Nymph	Adult	Nymph	Adult	Nymph	Adult	Nymph	Adult	Nymph	Adult	Nymph	Adult	Nymph	Adult	Nymph	Adult	
*Wsp*+/Samples	9/31	20/58	4/19	9/38	26/50	25/46	2/4	0/6	1/2	2/5	3/9	2/5	1/1	0/4	1/2	3/4	**108/284**
Total	29/89		13/57		51/96		2/10		3/7		5/14		1/5		4/6		**38.1%**

Based on the results, 47 out of 118 nymphs (40.2%) and 61 out of 166 adults (36.7%) were found positive in *wsp* PCR screening (Fig. 2, Table 1). Among positive samples, PCR products of 10 samples were selected randomly to perform sequencing. These samples carried *Wolbachia* DNA according to the BLAST process. The sequences obtained from this study have been deposited in GenBank under Accession Numbers KR706518 to KR706527. They are identical or highly similar (100% homology) to several *Wolbachia* sequences from *C. lectularius* deposited in GenBank, including isolates from Australia (AB475130, AB475131), Japan (AB475128, AB475129), and USA (DQ842459).

## Discussion

In recent years, several investigations have concentrated on *Wolbachia* infection among different arthropod hosts. These studies are important for several reasons: (i) *Wolbachia* species are important for studies targeting evolutionary processes due to their widespread distribution among different host populations, (ii) *Wolbachia* species can be used in studies focusing on biological and cellular processes because they lead to changes in the development and mitotic processes in their hosts, and (iii) due to the potential role of *Wolbachia* species in reducing host ability to reproduce and consequently decreasing the population size, they are an important part of biological control of vector/pest insect populations [2, 7, 17]. These reasons have thus encouraged researchers to investigate the occurrence and prevalence of *Wolbachia* in several different insects, including bed bugs.

For this purpose, studies conducted to survey *Wolbachia* infection among bed bug populations have used samples from nature as well as from laboratories. Rasgon and Scott [25] identified *Wolbachia* symbionts in laboratory colonized cliff swallow bugs (*Oeciacus vicarious Horvath*) and common bed bugs (*Cimex lectularius*) by PCR amplification and sequencing using *Wolbachia*-specific 16S rDNA and *FtsZ* genes. Their phylogenetic analyses indicated that *Wolbachia* infections in these two cimicid hosts form a monophyletic group, and the *Wolbachia* strains detected belong to the F clade. In the same way, Baldo et al. [1] carried out a multilocus sequence typing (MLST) study on 42 laboratory-reared bed bug specimens harboring single infection with different *Wolbachia pipientis* strains, using a specific set of markers, e.g. *wsp*, *FtsZ*, and 16S. Their results demonstrated that the *Wolbachia* strains isolated from their prospected *Cimex lectularius* belonged to the F supergroup. Four years later, Hosokawa et al. [14] characterized the bacterial symbionts of laboratory bed bug samples including two Japanese (TUA and TIH) and two Australian samples (SYDW and SYDL). They isolated *Wolbachia* species from each bed bug specimen individually and conducted PCR, cloning, and sequencing of a bacterial 16S rRNA gene fragment. They reported complete similarity for *Wolbachia* strains isolated from Japanese and Australian bed bugs.

All of the mentioned studies were carried out on bed bug samples from laboratory colonies. Endosymbiotic *Wolbachia* bacteria have previously been shown to infect laboratory colonies of the common bed bug, *Cimex lectularius*, but there is little information on the extent of infection in natural populations. One of the studies on the prevalence of *Wolbachia* infection among field collected samples was carried out by Sakamoto and Rasgon [29] on 39 bed bug samples from the United States and Zambia. They concluded that *Wolbachia* infections were prevalent in the populations with an infection rate of 83%–100% and there were no significant differences in infection frequency between geographic regions, between sexes, or between life stages (adult versus nymph). Recently, Siddiqui and Raja [33] assayed *Cimex lectularius* populations from different regions of India for *Wolbachia* infection using a PCR-based analysis. They reported an infection rate of over 75% for their prospected samples.

To our knowledge, the present study is the first epidemiological investigation carried out in France, as well as Europe, on field collected bed bugs in order to detect and identify *Wolbachia* strains and prevalence among samples that come from different regions of France.

The total rate of *Wolbachia* infection of bed bugs in this study is calculated as 38.2% which is lower than rates reported by Sakamoto and Rasgon [30] and by Siddiqui and Raja [33]. This value is, however, an underestimate compared to infection rates reported previously for *C. lectularius*. To date, no study has been conducted on *Wolbachia* infection in *C. lectularius* in France. However, analysis with more bed bug samples in terms of quantity and diversity from different biological (male/female, nymph/adult) and physiological (unfed/blood fed, parous/nulliparous) stages will enhance findings and provide a more accurate estimate of the *Wolbachia* infection rate in *C. lectularius* in France. Also, among positive cases, 10 samples were selected and sequenced randomly. The obtained sequences were edited, aligned, and blasted with GenBank database sequences to identify *Wolbachia* species. These sequences were identified as *Wolbachia* sp. and had 100% and 99% homology with other already known *wsp* sequences reported from *Cimex lectularius* (DQ842459 from the United States) and *Cimex japonicas* (AB508952 from Japan), respectively, which belong to the F supergroup strains of the *Wolbachia* genus. These sequences also had more than 95% homology with *Wolbachia pipientis* bacterium as a type strain of the *Wolbachia* genus, demonstrating the genetic variations and subdivision of the *Wolbachia* genus.

## Conclusion

This is the first epidemiological study of this kind carried out in France as well as Europe. This study allowed us to identify the prevalence of *Wolbachia* infection among several bed bug populations collected from different localities and to compare our findings with previously reported results in the literature. Our results demonstrate the occurrence of *Wolbachia* infection in both nymphs and adults of *C. lectularius*, although the infection rates were not similar. Based on sequencing results obtained from partial DNA amplification of the *wsp* gene, there was 100% homology with *Wolbachia wsp* sequences (previously associated with *C. lectularius*), reported from other regions, such as the United States and Japan*.*

## Competing interests

The authors declare that they have no competing interests.
